# Protein Modification Employing Non-Canonical Amino Acids to Prepare SUMOylation Detecting Bioconjugates

**DOI:** 10.3390/pharmaceutics14122826

**Published:** 2022-12-16

**Authors:** Alexander C. Williard, Hannah J. Switzer, Christina A. Howard, Rui Yin, Brent L. Russell, Ritwik Sanyal, Shaun Yu, Trinity M. Myers, Brian M. Flood, Oliver Kerscher, Douglas D. Young

**Affiliations:** 1 Department of Chemistry, William & Mary, Williamsburg, VA 23185, USA; 2 Department of Biology, William & Mary, Williamsburg, VA 23185, USA

**Keywords:** bioconjugates, non-canonical amino acids, SUMO, protein modification

## Abstract

Protein modification with non-canonical amino acids (ncAAs) represents a useful technology to afford homogenous samples of bioconjugates with site-specific modification. This technique can be directly applied to the detection of aberrant SUMOylation patterns, which are often indicative of disease states. Modified SUMO-trapping proteins, consisting of a catalytically inactive ULP1 fragment (UTAG) fused to the maltose-binding protein MBP, are useful reagents for the binding and labeling of SUMOylated proteins. Mutation of this UTAG fusion protein to facilitate amber suppression technologies for the genetic incorporation of ncAAs was assessed to provide a functional handle for modification. Ultimately, two sites in the maltose-binding protein (MBP) fusion were identified as ideal for incorporation and bioconjugation without perturbation to the SUMO-trapping ability of the UTAG protein. This functionality was then employed to label SUMOylated proteins in HeLa cells and demonstrate their enrichment in the nucleus. This modified UTAG-MBP-ncAA protein has far-reaching applications for both diagnostics and therapeutics.

## 1. Introduction

According to the National Cancer Institute of the NIH, prostate cancer is the most common cancer diagnosed in men, representing 14% of all new cancer cases in the United States [1]. Currently, the standard method of diagnosis is an assay that monitors the bloodstream concentration of prostate-specific antigen. However, the high rate of false positives often necessitates unnecessary biopsies [2]. Development of a more accurate, noninvasive method of prostate cancer diagnosis and surveillance would be a valuable clinical tool to decrease the rate of time-consuming and invasive procedures [3,4]. The difference in protein SUMOylation patterns between prostate cells and their healthy counterparts is a promising characteristic that could be leveraged to develop this diagnostic therapeutic [5]. Small ubiquitin-like modifier (SUMO) is a post-translational modification to proteins involved in a variety of cellular processes, including DNA replication, cell cycle regulation, and cell division [6]. In prostate cancer cells, many tumor suppressor proteins, including reptin, a protein involved in transcriptional regulation and chromatin remodeling, are hyper-SUMOylated [7,8]. Thus, the development of new methodologies to monitor SUMOylation can afford novel therapeutics and diagnostics. Ulp1 is a SUMO protease conserved in budding yeasts, including the stress-tolerant *K. marxianus*. Ulp1’s SUMO protease activity is required for SUMO precursor processing, as well as cleaving SUMO from modified proteins [9]. A SUMO-trapping fragment of Ulp1, UTAG, encompasses a catalytically inactive mutant fragment of the Ulp1 UD domain that can avidly bind to SUMO, but is incapable of cleavage [9,10]. Previous research has demonstrated that UTAG is capable of binding SUMO-1, SUMO-2, and SUMO-3; however, this study will primarily focus on association with SUMO-1 [9]. The ability to further modify and label the UTAG could provide an avenue for noninvasive prostate cancer diagnosis, as differences in SUMOylation between healthy and cancerous cells could be visualized within the cell.

**Figure 1:**
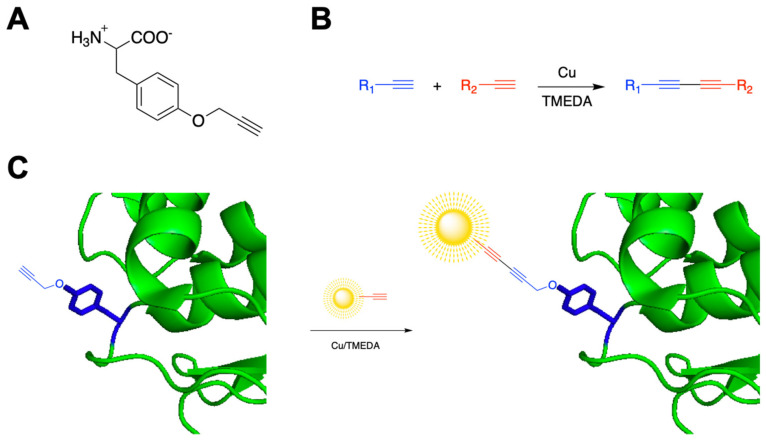
Bioconjugation strategy for preparation of modified UTAG diagnostic proteins. (**A**) The *p*-propargyloxyphenylalanine (*p*PrF) ncAA structure. (**B**) Representative Glaser–Hay conjugation of two terminal alkynes to generate a conjugated diyne. (**C**) Genetic incorporation of *p*PrF into UTAG followed by a Glaser–Hay bioconjugation with a fluorescent terminal alkyne to selectively label the protein.

Virtually all naturally occurring proteins in cells are comprised of the standard twenty canonical amino acids. Despite the great variety of protein functions, the utility of natural amino acids in some applications is diminished by their lack of chemical diversity. However, it is possible to chemically synthesize non-canonical amino acids (ncAAs) with a plethora of chemical functionalities, including terminal alkynes or azides [11,12,13]. Using already evolved amino-acyl tRNA synthetases, it is possible to charge a tRNA that recognizes the amber stop codon (TAG) with a ncAA of interest, allowing for incorporation of ncAAs at specific sites in the polypeptide sequence. Because ncAAs have unique functionality not found in any other protein in the cell, they afford a bioorthogonal handle for conjugation. Incorporation of a ncAA, such as *p*-propargyloxyphenyalanine (*p*PrF), introduces a terminal alkyne into UTAG and provides a terminal alkyne handle for modification (Figure 1) [14]. Moreover, alternative conjugation partners can be employed to deliver therapeutic molecules directly to disease state cells, preventing undesired side effects and toxicity [15,16,17]. Employment of ncAAs also provides control over the site of reactivity. This limits the formation of complex mixtures of conjugate valances and locations associated with conjugation at a canonical amino acid that potentially interfere with the function of the protein of interest [11].

Generally, bioconjugations are chemical reactions that couple a biomacromolecule, such as a protein, with another molecule. Bioconjugations can be difficult to perform, as chemical reactions often require conditions that are incompatible with biomolecules, such as organic solvents or high temperatures. Bioorthogonal chemistry involves a class of reactions that can occur efficiently in physiological settings without interfering with any of the normal cellular processes or reacting with any undesirable partner. One of these reactions is the Glaser–Hay coupling, which reacts two terminal alkynes to make a diyne linkage (Figure 1) [18]. The Glaser–Hay coupling can be performed under sufficiently mild conditions to conjugate the terminal alkyne of a protein containing a ncAA with a small molecule without causing degradation of the protein [19,20,21,22]. Provided conjugation does not significantly alter UTAG’s shape to make it incapable of binding SUMO, the Glaser–Hay coupling can render UTAG fluorescent by conjugating it to a fluorescent molecule. Ultimately, this research aims to optimize this bioconjugation to develop a novel diagnostic and/or therapeutic agent for the detection and treatment of the irregular SUMOylation that occurs under various disease states, including prostate cancer.

## 2. Materials and Methods

### 2.1. General

All reagents were obtained from Acros, USB, Biotium, Alfa Aesar, Sigma Aldrich, or Amresco and used without further purification unless noted. All PCR primers were obtained from IDT DNA Technologies Inc. (Coralville, IA, USA). The *p*PrF ncAA was prepared according to literature procedures [14]. Protein and DNA gels were analyzed using a Bio-Rad gel imager (Bio-Rad Molecular Imager Gel Doc XR+). Microscopy was performed on a BioRad Zoe inverted fluorescence microscope.

### 2.2. Mutagenesis

A thermocycler (Applied Biosystems 2720 Thermocycler) and the Q5 site-directed Mutagenesis kit (New England BioLabs, Ipswitch, MA, USA) were employed to perform site-directed mutagenesis to introduce the TAG codon. A pET plasmid harboring an MBP-UTAG fusion was diluted 1:10, 1:100, and 1:1000. The PCR reaction mixture contained one of the dilutions of the plasmids (1 μL), forward primer (10 μM, 1.25 μL), reverse primer (10 μM, 1.25 μL), Q5 Hot Start Master Mix (12.5 μL), and Milliq water (9 μL). The reaction mixture was subjected to the following heating protocol: 98 °C (30 s); eighteen cycles of melting (98 °C, 10 s), annealing (60–70 °C, 30 s), and extension (72 °C, 8 min); a final extension period (72 °C, 10 min); and then held at 4 °C. The annealing temperature was matched to the annealing temperature of the primers for each reaction. Using the Q5 Site-Directed Mutagenesis Kit, the parent plasmid was digested, and the mutant DNA was circularized. The reaction (5 μL) was transformed into DH5α competent *Escherichia coli* (New England Biolabs) via heat shock. The transformed cells were plated (15, 50, 120 µL) on agar containing (1 mg/mL) ampicillin and incubated overnight at 37 °C. The cells were miniprepped using an IBI High Speed Plasmid Miniprep Kit (VWR). The resulting plasmids were analyzed for successful insertion of the TAG mutation by sequencing at the William & Mary Molecular Core Facility (Williamsburg, VA, USA).

### 2.3. Protein Expression and Purification

DH5α *E. coli* competent cells containing the rare tRNA plasmid pRIL were purchased from VWR International (Radnor, PA, USA). These electrocompetent cells were co-transformed with pULTRA-*p*CNF synthetase plasmid (2 μL) and mutagenized UTAG fusion protein plasmid containing the TAG codon (2 μL) using an Eppendorf electroporator. These cells were plated onto LB-agar containing 50 mg/mL ampicillin, 34 mg/mL chloramphenicol, and 30 mg/mL spectinomycin and incubated at 37 °C for 16 h. A single colony from this plate was selected and used to inoculate LB media containing the 3 antibiotics at 37 °C overnight. This starter culture was then used to make an expression culture in 25 mL LB media, beginning at an OD_600_ of 0.1 and induced at OD_600_ between 0.7 and 0.8. The expression was induced with 25 μL of 1 mM IPTG (25 μL), 20% arabinose (25 μL), and 100 mM p-propargyloxyphenylalanine (*p*PrF; 250 μL). The cells were incubated at 37 ℃ for 16 h and then pelleted by centrifugation (5000 rpm, 10 min, 4 °C). The LB media was decanted, and the remaining pellet was placed in a −80 °C freezer for 20 min. The mutant protein was purified from the pellet using commercially available Ni-NTA spin columns (Biotium, Fremont, CA, USA), according to the manufacturer’s protocols. The quality of protein expression was assessed by 10% SDS-PAGE and a BCA assay (Biotium). The protein was then buffer exchanged into phosphate buffered saline (PBS, pH 7) using 10 k MWCO spin columns (Corning, Corning, NY, USA).

### 2.4. Glaser–Hay Bioconjugation

The reactants were added to a microcentrifuge tube in the following order: copper iodide (5 μL, 500 mM) was added to N,N,N′,N′-Tetramethylethylenediamine (TMEDA; 500 mM, 5 μL), and the complex was allowed to form at room temperature for 15 min. Next, UTAG-*p*PrF (0.335 mg/mL, 15 μL), alkyne fluorophore (Fluor-488-Alkyne, 1 mM, 10 μL), catalyase (10 mg/mL, 10 μL), and PBS (pH 7.6, 8 μL) were added to the tube. The reaction was incubated at 22 °C for 4 h then transferred to a 10 kDa molecular weight cutoff column, washed with PBS Buffer (8 × 80 μL), and centrifuged at 13,200 rpm for 2 min. The reactions were then analyzed via SDS-PAGE (10%) and imaged for fluorescence prior to being stained with Coomassie Blue. Gels were destained in acetate/methanol and re-imaged to confirm protein identity and purity.

### 2.5. Pull down Assay on Fluorescent Labeled UTAG

Beads containing immobilized SUMO-1 (Boston Biochemical, rhSUMO-1 beads; 25 μL) were washed with Sumo Protease Buffer (SPB; 50 mM Tris-HCl, pH 8.0, 0.2% NP-40, 150 mM NaCl, 5 mM TCEP; 1 mL). Following the initial wash, SPB (900 μL), TCEP (50 mM, 100 μL), and UTAG variant protein (30 μL) were added to the washed beads and rotated for 30 min at room temperature. The beads were then washed with SPB (3 × 1 mL) by pelleting the beads and pipetting away the supernatant SPB. The beads were resuspended in PBS (20 μL) and analyzed using a Bio-Rad gel imager (BioRad Molecular Imager Gel Doc XR+). The resin was also imaged by transferring the resin to a 96-well plate and adding PBS (50 μL). The resin was then analyzed for fluorescence on a plate reader (Biotek Synergy HT Microplate Reader, Winooski, VT, USA). The protocol was set to measure the fluorescence endpoint after 5 s of shaking, at an excitation wavelength of 485 nm and an emission wavelength of 528 nm. Finally, beads in the plate were also analyzed by fluorescence microscopy on a BioRad Zoe inverted fluorescent microscopy (Hercules, CA, USA) to confirm the presence of the immobilized fluorescent protein. Maltose binding was assessed using the same procedure, substituting amylose resin/beads (New England BioLabs) and amylose column buffer (20 mM Tris-HCl, pH 7.4, 0.2 M NaCl, 1 mM EDTA).

### 2.6. Plate Pull down Assay

Poly-L-lysine (200 μL, 0.1% *w*/*v* in water) was added to 2 wells on a 96-well plate and incubated at room temperature for 5 min. The wells were emptied and allowed to dry for 5 min, then washed once with SPB (300 μL). SUMO-1 was added to the wells and the plate was rocked for 30 min. The wells were washed 3 times with SPB (300 μL), and 1% (*w*/*v*) BSA in SPB (200 μL) was added to the wells. The plate was rocked at 4 °C for 16 h, then the wells were washed with SPB. The variant UTAG protein (10 μL) was added to 90 μL of SPB containing 5 mM TCEP. The plate was rocked at 4 °C for 1 h. The wells were washed 3 times with SPB containing 5 mM TCEP (300 μL) and analyzed on a plate reader (Biotek Synergy HT Microplate Reader). The protocol was set to measure the fluorescence with an excitation wavelength at 485 nm and the emission wavelength at 528 nm.

### 2.7. Mammalian Cell Detection of SUMO with Bioconjugate

The assay was performed as described in the literature [23]. HeLa cells (ATTC) were seeded on 22 mm round cover slides placed inside 6-well plates in DMEM and incubated in a CO_2_ incubator at 37 °C until 70–80% confluent. The cells were then washed with DPBS (Dulbecco’s phosphate-buffered saline; 1 mL) and aspirated. A fresh solution of 4% PFA (paraformaldehyde) in DPBS was prepared and 2 mL was added to each well, followed by a 20 min incubation at room temperature to fix the cells. The cells were then washed with DPBS (3 × 1 mL), with a 5 min incubation per wash. After the final aspiration of the media, 0.1% Triton X-100 in DPBS (1 mL) was added to permeabilize the cells, which were incubated for 1 h and then washed again with DPBS (3 × 1 mL). Cells were then incubated at room temperature with 0.1 M Glycine-HCL (500 µL; pH 2.0) for 30 s, then immediately neutralized with 10× SPB (500 mM Tris-HCl, pH 8.0, 2% NP-40, 1.5 M NaCl; 500 µL). A final wash was performed with DPBS (3 × 1 mL) prior to incubation with UTAG variant (1 µg in 1 mL DPBS) for 1 h at room temperature. The coverslips were then washed with DPBS (3 × 200 µL) and inverted onto a microscope slide with DAPI staining mounting medium. The slides were next imaged on a BioRad Zoe inverted fluorescence microscope for both AlexaFluor-488 and DAPI to identify localization of the UTAG variant.

## 3. Results and Discussion

### 3.1. UTAG Mutagenesis and Assessment

**Figure 2:**
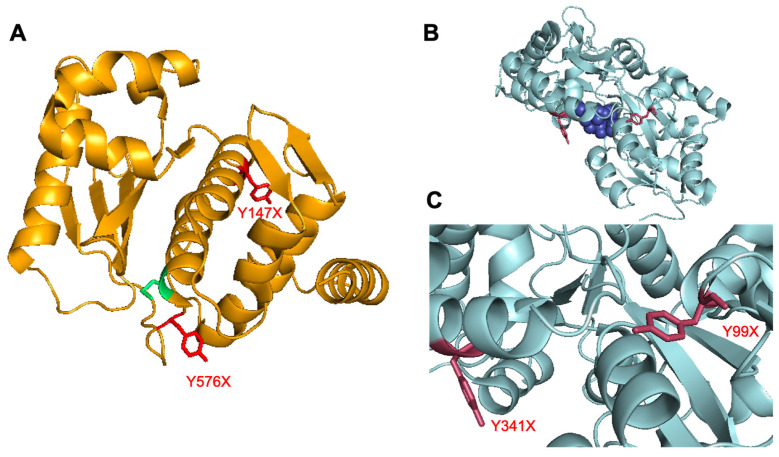
Crystal structures with surface-exposed tyrosine residues for ncAA incorporation. (**A**) Crystal structure of *S. cerevisiae* ULP1 protein. UTAG protein is generated via mutation of catalytically active cys residue (green). Sites of surface exposed tyr residues (red) were identified as ideal locations for ncAA incorporation (PDB 2HKP) [27]. (**B**) Crystal structure of maltose-binding protein (MBP) that is expressed as a fusion with UTAG (PDB 3RLF) [28]. The active site is displayed with an amylose bound (dark blue). (**C**) Closer image of the two tyr residues (red) selected for ncAA incorporation. The first Y99 is in a loop structure with significant conformational freedom, and the Y341 is projected outward from an α-helix.

Initial studies investigated two isoforms of UTAG, one from *Saccharomyces cerevisiae* (*Sc*) and one from *Kluyveromyces marxianus* (*Km*), with each harboring the key mutation that renders it catalytically inactive [9]. The sequence differences between the two isoforms has been reported in the literature, but both are able to bind various isoforms of SUMO [9]. The *Km* UTAG is a heat-stable protein and, thus, may exhibit more desirable properties for final employment as a therapeutic or diagnostic. Both the *Sc* UTAG and the *Km* UTAG were expressed as fusion proteins with maltose-binding protein (MBP), as this fusion has been previously demonstrated to further stabilize the protein. Based on crystal structure analysis of the two isoforms, key surface-exposed tyrosine residues were selected for genetic incorporation of the ncAA: Y147 and Y576 (Figure 2A). Site-directed mutagenesis of these residues in both isoforms was performed to introduce the TAG codon for transcriptional incorporation of a ncAA at each site. Following sequencing to confirm successful mutagenesis, the UTAG plasmid was co-transformed with a pEVOL-*p*CNF plasmid that provides the translational machinery for amber codon suppression with ncAAs. Moreover, this particular aminoacyl-tRNA synthetase/tRNA pair has been demonstrated to be polyspecific and, thus, able to incorporate several different ncAAs into the protein based on which ncAA is added to the expression media [24]. This promiscuity facilitates a greater degree of modularity, providing the ability to introduce different functional handles into the protein via alternative reactions (e.g., *p*-azidophenylalanine for 1,3-dipolar cycloadditions [25] or *p*-acetylphenylalanine for oxime couplings [26]).

**Figure 3:**
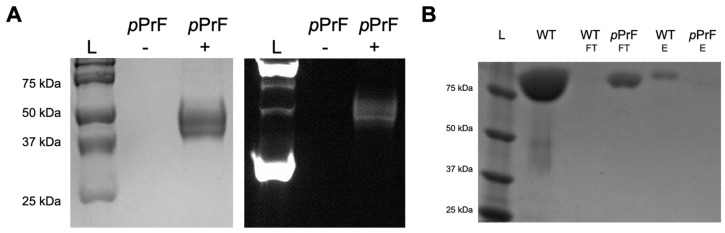
Methodology validation of UTAG expression with *p*PrF ncAA. (**A**) Initial attempts at expression of the Sc Y147TAG mutant. Similar results were observed with all mutation sites in both *Sc* and *Km* expressions. While full-length expressed protein was expected around 84 kDa, a significantly shorter protein of ~40 kDa was produced (Coomassie stain gel on left). The expression did appear to contain *p*PrF as no protein product was observed in the absence of *p*PrF addition (−), but protein was detected with the addition of the ncAA (+). Incorporation of the ncAA was further confirmed as the sample was also subjected to Glaser–Hay bioconjugation with AlexaFluor-488, resulting in the covalent modification of the protein and a fluorescent band (right gel; +lane). L = protein ladder. (**B**) Following modification of expression conditions, full-length UTAG was produced that harbored the *p*PrF ncAA. The protein was then assayed for SUMO binding with a pull-down bead-based assay. Both the flow-through (FT) and elution (E) of the assay were observed by SDS-PAGE. For the wild-type protein, very little protein was observed in the flow-through (WT-FT), suggesting SUMO binding, which was confirmed by the presence of UTAG in the elution (WT-E). In the case of the *Km* Y576TAG mutant (and all other mutants), the UTAG protein was only observed in the flow-through (*p*PrF-FT) and not in the elution (*p*PrF-E). L = protein ladder; WT = wild-type control.

Expression of both the *Sc* and *Km* mutants initially resulted in truncated proteins; however, all were of similar molecular weight, indicating that truncation was not a result of failure to suppress the amber stop codon. If translation had halted in response to the TAG codon, the two mutation sites should have produced significantly different fragment sizes. Moreover, Glaser–Hay coupling with an AlexaFluor-488 alkyne resulted in fluorescent product, indicating the successful incorporation of the alkynyl ncAA (Figure 3A). Consequently, it was concluded that the protein truncation was instead due to differences in codon bias in the *E. coli* host, resulting in a deficit of rare tRNAs and inability to translate full-length protein. To address this tRNA deficit, expressions were shifted to DH5 α cells containing a pRIL plasmid to facilitate expression of rare tRNAs. Due to the chloramphenicol marker of this pRIL plasmid, introduction of the translational machinery was shifted to a pULTRA-pCNF system that confers spectinomycin resistance, and expressions were conducted in the presence of three antibiotics. The migration to the new expression platform was successful, and full-length protein with the *p*PrF ncAA was obtained. This protein was then coupled to a fluorophore using the previously developed bioorthogonal Glaser–Hay reaction [19,20,21,22].

With the modified UTAG expressed and fluorescently labeled for both mutation sites in *Sc* and *Km*, the UTAG was assessed for SUMO binding using two assays. In one, a plate pull-down assay was performed where SUMO was surface immobilized in a 96-well plate and incubated with the modified UTAG proteins, followed by fluorescence detection [10]. The second involved a bead pull-down assay where immobilized SUMO resin was treated with the fluorescent UTAG, washed, and then eluted to be analyzed by both fluorescence spectroscopy and SDS-PAGE. Unfortunately, SUMO binding was not observed in either of the assays for any of the mutants. The bead pull-down assay was validated by the binding of wild-type UTAG that did not contain the ncAA or the fluorescent label (Figure 3B). This outcome suggests that the sites that were selected for ncAA incorporation may have caused some form of structural perturbation that prevents SUMO binding. Additional expressions with *p*-azidophenylalanine (*p*AzF) in place of *p*PrF had similar binding results, indicating that it was not simply the ncAA structure alone that inhibited binding. These results necessitated a re-evaluation of the approach, but they remain a useful illustration of the importance of mutagenesis sites when making protein modifications with ncAAs.

### 3.2. Maltose-Binding Protein Mutagenesis and Evaluation

**Figure 4:**
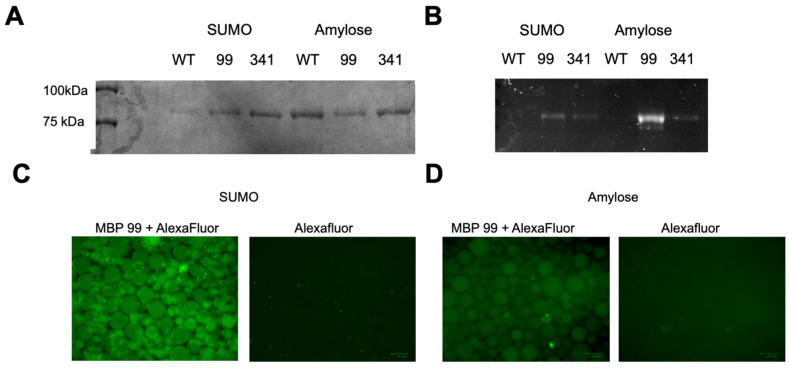
Bead-based assessment of *Km* UTAG-MBP constructs fluorescently labeled by ncAA incorporation and bioconjugation. (**A**) SDS-PAGE analysis of both *Km* UTAG-MBP mutants following incubation with either SUMO resin for UTAG binding or Amylose resin for MBP binding. Elutions of all assays were analyzed, and binding is observed in both the Y99 and Y341 mutants, as well as the wild-type UTAG-MBP (WT). (**B**) SDS-PAGE analysis of fluorescence detected following the bead pull-down assays. In both resins, fluorescence is observed only for the AlexaFluor-488-modified variants. (**C**) Fluorescence microscopy of SUMO beads incubated with *Km*-UTAG-341-*p*PrF/AlexaFluor-488 conjugates versus beads incubated only with the AlexaFluor-488 (scale bar = 100 mM). (**D**) Fluorescence microscopy of Amylose beads incubated with *Km*-UTAG-99-*p*PrF/AlexaFluor-488 conjugates versus beads incubated only with the AlexaFluor-488. All assays were conducted in triplicate.

Because of the unsuccessful direct modification of the UTAG protein, we next investigated the modification of the maltose-binding protein (MBP) fusion partner that confers additional stability to the UTAG for therapeutic and diagnostic applications. Moreover, given that MBP is a common fusion tag employed in molecular biology, it affords a more general approach to the modification of any protein that employs MBP as a purification tag (Figure 2B). After crystal structure analysis of MBP, two surface-exposed tyrosine residues were selected and mutated for ncAA expression: Y99 and Y341 (Figure 2C). As previously described, the *Km* UTAG-MBP fusion proteins were expressed in the DH5-α (pRIL) cells with *p*PrF to produce full-length protein harboring the desired ncAA. Following expression and purification, the protein was modified via Glaser–Hay bioconjugation with the AlexaFluor-488 alkyne. After successful expression and conjugation was verified by SDS-PAGE, the two *Km* UTAG mutants were assayed for SUMO binding, which was lost with the direct UTAG modification. Initially, the earlier-described bead pull-down assay was performed to determine if the ncAA again affected SUMO binding. With these bioconjugated UTAG-MBP mutant proteins, SUMO binding was observed with only protein detected in the elutions (Figure 4A). This binding was further confirmed by observing the in-gel fluorescence, where the fluorescent bands corresponded to both of the mutants and not the unlabeled wild-type protein (Figure 4B). While detection of SUMOylation was the primary aim, we also were interested to see if the protein modification affected the binding of the MBP, as it was the direct site of modification. Gratifyingly, a comparable bead-based assay substituting amylose beads for the SUMO beads yielded a similar result with protein only observed in the elutions and not in the flow-through (Figure 4). This indicates that maltose binding is not inhibited by placement of the ncAA in either the 99 or the 341 position, and it can still be employed as a modified affinity tag. Binding was also confirmed in both sets of washed resins by fluorescence microscopy (Figure 4C,D). Beads incubated with fluorophore alone did not exhibit non-specific binding; thus, the consequent fluorescence derives from the association of the modified UTAG-MBP with the corresponding SUMO or amylose resins. The combination of these results suggests that a modified and functional fluorescent construct has been developed for the detection of SUMOylation.

### 3.3. Assessment of SUMO Binding in Mammalian Cells

**Figure 5:**
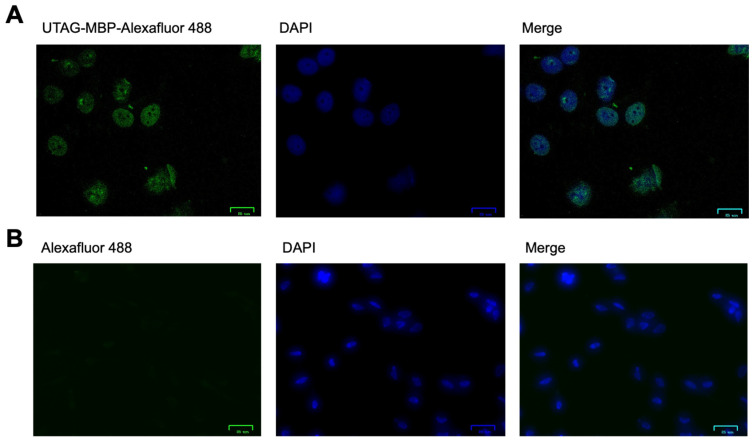
Fluorescence microscopy of *Km*-UTAG-MBP binding to SUMO in HeLa cells. (**A**) HeLa cells incubated with the *Km*-UTAG-99-*p*PrF/AlexaFluor-488 construct demonstrated fluorescence associated with AlexaFluor488 (green) and DNA DAPI stain (blue). Co-localization is observed in the merged image (scale bar = 25 υM) (**B**) HeLa cells incubated with only AlexaFluor-488 did not display AlexaFluor-488 fluorescence (far left); however, they did exhibit DNA DAPI fluorescence (blue; middle). Only the DAPI is observable in the merged image (far right; scale bar = 25 υM).

With fluorescent SUMO-detecting protein conjugates in hand, we next sought to demonstrate their effective labeling of SUMOylation in a more practical scenario. Given that it is documented that nuclear SUMOylation is common in HeLa cells, we performed a proof-of-concept experiment to illustrate the functionality of these constructs. HeLa cells were grown to confluency in DMEM on a cover slip for 48 h at 37 °C, the media was aspirated, and the cells were fixed according to literature precedence to allow conjugate binding [23]. Cells were then incubated with either the *Km*-UTAG-99-*p*PrF/AlexaFluor-488 construct or AlexaFluor-488 alkyne alone, then washed, stained with DAPI to identify nuclei, and imaged with fluorescence microscopy (Figure 5). As SUMOylation occurs in high levels in the nuclei, it was hypothesized that UTAG association should be enriched in the nuclear compartment. The microscopy results were in accordance with this hypothesis, as AlexaFluor-488 fluorescence was co-localized with the DAPI nuclear stain. Additionally, no background binding of the AlexaFluor-488 was observed, indicating that the fluorescence in the bioconjugate-treated cells is a result of SUMO-UTAG association.

## 4. Conclusions

In conclusion, ncAAs were employed to modify a SUMO-binding construct that can detect SUMOylation levels within cells. This *Km*-UTAG-MBP bioconjugate exploits the novel functionality introduced by ncAAs to further expand protein function. While fluorescent constructs could be easily generated via genetic fusions of fluorescent proteins, our approach makes a minimal perturbation to introduce a small molecule fluorophore in a site-specific fashion, permitting an advantageous advancement to current technologies. Moreover, the unique chemical functionality introduced in this methodology allows for biorthogonal couplings to be performed, which exert a high level of control over the reaction, limiting it to a single residue and a single modification. Further application of SUMO binding is underway as we assess its potential to be employed as a diagnostic agent and as a means of quantifying SUMOylation within cells. Along these lines, we are currently investigating the quantification of SUMOylation between cancerous cells and healthy cells to develop a sensitive quantitative assay for differentiation between healthy and cancerous cells. Moreover, the construct could ultimately be utilized as a therapeutic delivery system by way of conjugating a drug or therapeutic agent for cellular delivery in place of the fluorophore. Finally, due to the modification of the MBP fusion partner domain instead of directly on the protein of interest, this system can function more modularly and be utilized to label and/or conjugate to a variety of proteins that are expressed with the modified MBP tag. Therefore, this approach to protein modification has resulted in a valuable construct with far-reaching therapeutic and diagnostic applications.

## Data Availability

Not applicable.
